# Association of serum asprosin with metabolic dysfunction-associated fatty liver disease in older adult type 2 diabetic patients: a cross-sectional study

**DOI:** 10.1186/s12902-024-01560-1

**Published:** 2024-03-04

**Authors:** Junfang Cui, Yunfeng Liu, Mina Li, Jianhong Yin, Jing Yang, Linxin Xu

**Affiliations:** 1 Department of Geriatrics, The First Hospital of Shanxi Medical University, Taiyuan, China; 2 Department of Endocrinology, The First Hospital of Shanxi Medical University, Taiyuan, China; 3 Shanxi Medical University, Taiyuan, China

**Keywords:** Metabolic dysfunction-associated fatty liver disease, Type 2 diabetes mellitus, Blood biomarker, Asprosin, Screening

## Abstract

**Background:**

To explore the association of serum asprosin levels with metabolic dysfunction-associated fatty liver disease (MAFLD) in older adults with type 2 diabetes mellitus (T2DM).

**Methods:**

The cross-sectional study enrolled patients ≥ 65 years old diagnosed with T2DM at two community health service centers between November 2019 and July 2021. Logistic regression was applied to analyze the influencing factors of MAFLD.

**Results:**

Totally 219 cases were included. Compared with diabetic individuals without MAFLD (*n* = 105), diabetics with MAFLD (*n* = 114) had younger ages, higher body mass index values, shorter time from T2DM diagnosis, increased waist-to-hip ratios, elevated triglycerides, reduced high-density lipoprotein cholesterol (HDL-C), elevated alanine aminotransferase (ALT), elevated γ-glutaryl transferase, elevated fasting insulin, and elevated HOMA-IR (all *P* < 0.05). Serum asprosin levels were elevated in diabetics with MAFLD in comparison with the non-MAFLD group (291.71 ± 73.69 vs. 255.24 ± 82.52 pg/ml, *P* = 0.001). Multivariable analysis revealed, after adjusted for age, time from T2DM diagnosis, HDL-C, and ALT, serum asprosin level (OR = 1.006, 95%CI: 1.001–1.010, *P* = 0.014) were independently associated with MAFLD in T2DM.

**Conclusions:**

High asprosin level are associated with MAFLD in older patients with T2DM, after adjusted for age, time from T2DM diagnosis, WHR, TG, HDL-C, ALT, GGT, FINS, and HOMA-IR.

## Background

Metabolic dysfunction-associated fatty liver disease (MAFLD) results from fatty liver changes unrelated to alcohol intake [1–3]. The reported prevalence of MAFLD is 24–45% [1–3]. MAFLD has significant associations with insulin resistance, obesity, weight gain, and type 2 diabetes mellitus (T2DM). Metabolic dysfunction-associated steatohepatitis (MASH) is the advanced stage of MAFLD, and MASH, in turn, can progress to cirrhosis [1–3]. Of all patients with T2DM, 76% and 56% have MAFLD and MASH, respectively [1–3]. Progression from MASH to cirrhosis may be uncommon but could be promoted by obesity and diabetes [4]. MASH is associated with elevated mortality compared with MAFLD [5]. T2DM was affecting 9% and 7.9% of adult men and women in 2014 globally, respectively [6], but showed a prevalence of 14.6% in 2017–2018 in the United States of America (USA) [7] and 12.4% in 2018 in China [8]. The detrimental complications of T2DM include cardiovascular disease, neuropathy, nephropathy, retinopathy, and even mortality [9–13]. Patients with T2DM and MAFLD require very aggressive management to prevent T2DM complications and the progression of MAFLD to MASH, but caution must be taken to avoid overtreatment and drug side effects in T2DM patients without MAFLD [14–16].

Asprosin represents a novel protein hormone produced by the white fat tissue, which promotes glucose release by the liver. In T2DM, asprosin levels are elevated and positively correlated with insulin resistance and may serve as a biomarker to predict T2DM onset and monitor treatment effects [17]. Asprosin and adiponectin are elevated in obese children with MAFLD [18], and their combination is considered a diagnostic marker of MAFLD [19].

Indeed, MAFLD diagnosis relies on imaging and histopathology. Biopsy remains the gold standard for MAFLD diagnosis but represents an invasive tool not easily accepted by patients [20] and cannot be performed routinely for screening purposes [21]. Ultrasound is considered the first-line imaging modality for MAFLD, with a sensitivity of 60–94% and a specificity of 84–95%, both of which increase with MAFLD severity [21]. Computed tomography (CT) is more expensive and involves radiation, which is not feasible for screening or follow-up [1, 21]. Magnetic resonance imaging (MRI) constitutes the most accurate imaging tool for MAFLD detection but is even more expensive than CT and less readily available [1, 21]. Previous studies have indicated that various blood markers, e.g., alanine aminotransferase (ALT), aspartate aminotransferase (AST), γ-glutaryl transferase (GGT), triglycerides (TG), and C-reactive protein (CRP), are elevated in MAFLD, albeit with low sensitivity and specificity [1, 21, 22]. Hence, asprosin could be a diagnostic biomarker for MAFLD, but its association with MAFLD in the context of T2DM is poorly described.

Therefore, this study explored the association of asprosin with MAFLD in older adults with T2DM. The findings could help improve the clinical management of MAFLD.

## Methods

### Study design and patients

This cross-sectional study included elderly individuals diagnosed with T2DM at Zhuoma Community Health Service Station and Chengbei Xijie Community Health Service Center in Changzhi City (Shanxi Province) from November 2019 to July 2021. This study had approval from the Ethics Committee of the First Hospital of Shanxi Medical University (2019 [K056]). The study kept patient data confidential and complied with the Declaration of Helsinki.

Inclusion criteria were (1) age ≥ 65 years, (2) meeting the 1999 World Health Organization (WHO) diagnostic criteria for T2DM [23], and (3) available asprosin measurements and clinical data (detailed in the “Data collection and definitions” section below). Exclusion criteria were (1) acute complications of T2DM (e.g., hypoglycemia, diabetic ketoacidosis, and hyperosmolar hyperglycemic syndrome), (2) tumors, (3) acute heart failure, (4) acute renal failure, (5) steroid-induced diabetes, or (6) pulmonary infection.

Cases were assigned to the MAFLD and non-MAFLD groups. A diagnosis of MAFLD was made based on the available literature [24]: (1) weekly alcohol intake below 140 and 70 g in men and women, respectively, (2) no primary or secondary cause of fatty liver, and (3) liver biopsy revealing fatty liver. Since not all patients accepted liver biopsy, MAFLD could be clinically diagnosed [24]: (1) idiopathic fatty liver according to liver ultrasound or (2) idiopathic serum ALT, AST, and/or GGT elevations for ≥ 6 months in patients with metabolic syndrome.

### Data collection and definitions

Demographic (age and sex), clinical (BMI, waist-to-hip ratio [WHR], drinking history, time from T2DM diagnosis, systolic [SBP] and diastolic [DBP] blood pressure), and biochemistry (total cholesterol [TC], TG, high-density lipoprotein cholesterol [HDL-C], low-density lipoprotein cholesterol [LDL-C], ALT, AST, GGT, creatinine [CRE], glycated hemoglobin [HbA1C], fasting plasma glucose [FPG], estimated glomerular filtration rate [eGFR], 2-hour postprandial plasma glucose [2hPG], fasting insulin [FINS], homeostasis model assessment of insulin resistance [HOMA-IR], fasting C-peptide [FC-P], and asprosin) data were collected from patient charts. HOMA-IR was calculated as (glucose×insulin)/22.5 (SI measurements) [25].

### Statistical analysis

It was a retrospective study, and we included all eligible patients during the study period. Nevertheless, we use α = 0.05, power of 90%, Z_α_=1.96, Z_power_=1.28, σ = 0.43, and δ = 0.3 and the formula below, a minimum of 27 patients were required for each group.

$$ n = \frac{{{{(1.96 + 1.28)}^2}*2*0.43*0.43}}{{{{(0.3)}^2}}} \approx 27$$

Data analysis was carried out with SPSS 22.0 (IBM Corp., Armonk, NY, USA). Continuous data were assessed for normality by the Kolmogorov-Smirnov test. Those with normal distribution were presented as mean ± standard deviation, and between-group comparisons used the independent Student’s t-test. Non-normally distributed continuous data were expressed by median (25th percentile, 75th percentile) and compared between groups by the Mann-Whitney U test. Categorical variables were expressed by number (percentage) and compared by the chi-square test. Factors associated with MAFLD were determined by logistic regression analysis. The multivariable model included parameters with *P* < 0.05 in univariable analysis using the Enter method. The associations of serum asprosin level with various factors were assessed by Spearman analysis. Two-tail *P* < 0.05 indicated statistical significance.

## Results

Totally 219 patients were analyzed. In comparison with diabetics without MAFLD (*n* = 105), patients in the MAFLD group (*n* = 114) had younger ages (median, 71.0 vs. 72.0 years, *P* = 0.045) and higher BMI values (median, 26.1 vs. 23.8 kg/m^2^, *P* < 0.001). In addition, the MAFLD group showed a shorter time from T2DM diagnosis (median, 10.0 vs. 14.0 years, *P* < 0.001) and larger WHRs (median, 0.92 vs. 0.90, *P* = 0.018). Regarding biochemistry parameters, MAFLD cases had higher TG levels (median, 1.57 vs. 1.26 mM, *P* < 0.001), decreased HDL-C (median, 0.97 vs. 1.04 mM, *P* = 0.002), elevated ALT (median, 17.0 vs. 14.0 IU/L, *P* = 0.001), elevated GGT (median, 24.0 vs. 18.0 IU/L, *P* < 0.001), elevated FINS (median, 4.96 vs. 2.84 mIU/L, *P* < 0.001), and elevated HOMA-IR (median, 1.71 vs. 1.07, *P* < 0.001). Serum asprosin levels were elevated in diabetics with MAFLD compared with those without MAFLD (291.71 ± 73.69 vs. 255.24 ± 82.52 pg/ml, *P* = 0.001) (Table 1).

**Table 1:** Patient characteristics

Clinical data	Non-MAFLD group (*n* = 105)	MAFLD group (*n* = 114)	*P*
Age, years	72.0 (69.0, 75.0)	71.0 (68.0, 74.0)	0.045
Sex			0.699
Male	46 (43.8%)	47 (41.23%)	
Female	59 (56.2%)	67 (58.77%)	
BMI, kg/cm^2^	23.8 (21.5, 26.0)	26.1 (24.2, 27.8)	< 0.001
Time from T2DM diagnosis, years	14.0 (9.5, 20.0)	10.0 (5.0, 15.0)	< 0.001
WHR	0.90 (0.89, 0.96)	0.92 (0.90, 1.00)	0.018
Drinking history	31 (29.5%)	32 (28.1%)	0.812
Diabetes history	15 (14.3%)	16 (14.0%)	0.958
SBP (mmHg)	130 (120, 142.5)	130 (120, 140)	0.615
DBP (mmHg)	80 (70, 80)	80 (70, 80)	0.252
TC (mM)	4.72 ± 1.01	4.60 ± 1.04	0.412
TG (mM)	1.26 (0.92, 1.64)	1.57 (1.21, 2.22)	< 0.001
HDL-C (mM)	1.04 (0.89, 1.35)	0.97 (0.82, 1.09)	0.002
LDL-C (mM)	2.85 ± 0.86	2.81 ± 0.95	0.733
ALT (IU/L)	14.00 (10.00, 19.50)	17.00 (13.00, 27.25)	0.001
AST (IU/L)	17.00 (13.50, 21.00)	18.00 (14.00, 24.00)	0.223
GGT (IU/L)	18.00 (14.00, 25.50)	24.00 (18.00, 34.25)	< 0.001
Scr (µmol/L)	61.00 (53.50, 73.50)	60.00 (51.75, 71.00)	0.406
Asprosin (pg/ml)	255.24 ± 82.52	291.71 ± 73.69	0.001
eGFR (mL/min/1.73 m^2^)	128.78 (106.66, 154.52)	131.28 (114.72, 158.91)	0.612
HbA1C (%)	8.50 (7.15, 10.55)	8.95 (7.78, 10.4)	0.233
FPG (mM)	7.65 (5.98, 9.77)	7.41 (6.50, 9.86)	0.903
2hPG (mM)	12.78 ± 4.59	13.20 ± 3.79	0.463
FINS (mIU/L)	2.84 (2.00, 4.83)	4.96 (3.43, 7.24)	< 0.001
HOMA-IR	1.07 (0.6, 1.69)	1.71 (1.29, 2.45)	< 0.001
FC-P (nM)	1.81 (1.17, 2.43)	1.94 (1.12, 2.48)	0.614

All data are median (25th, 75th), mean ± standard deviation, or n (%)

MAFLD, metabolic dysfunction-associated fatty liver disease; BMI, body mass index; WHR, waist-hip ratio; SBP, systolic blood pressure; DBP, diastolic blood pressure; TC, total cholesterol; TG, triglycerides; HDL-C, high-density lipoprotein cholesterol; LCL-D, low-density lipoprotein cholesterol; ALT, alanine aminotransferase; AST, aspartate aminotransferase; GGT, γ-glutaryl transferase; Scr, serum creatinine; eGFR, estimated glomerular filtration rate; HbA1c, glycated hemoglobin; FPG, fasting plasma glucose; 2hPG, 2-hour postprandial glucose; FINS, fasting insulin; HOMA-IR, homeostasis model assessment of insulin resistance; FC-P, fasting C-peptide

The multivariable analysis is summarized in Table 2. After adjusted for age (OR = 0.923, 95%CI: 0.853–0.999; *P* = 0.046), time from T2DM diagnosis (OR = 0.919, 95%CI: 0.876–0.965; *P* = 0.001), HDL-C (OR = 0.089, 95%CI: 0.018–0.429; *P* = 0.003), and ALT (OR = 1.042, 95%CI: 1.007–1.079; *P* = 0.018), serum asprosin levels (OR = 1.006, 95%CI: 1.001–1.010; *P* = 0.014) were independently associated with MAFLD in T2DM.

**Table 2:** Multivariable analysis of factors associated with type 2 diabetes mellitus accompanied by MAFLD.

	OR (95% CI)	*P*
Age	0.923 (0.853, 0.999)	0.046
BMI	1.066 (0.996, 1.141)	0.064
Time from T2DM diagnosis	0.919 (0.876, 0.965)	0.001
WHR	4.432 (0.027, 737.124)	0.568
TG	1.662 (0.999, 2.766)	0.050
HDL-C	0.089 (0.018, 0.429)	0.003
ALT	1.042 (1.007, 1.079)	0.018
GGT	0.990 (0.974, 1.006)	0.199
Serum asprosin levels	1.006 (1.001, 1.010)	0.014
FINS	1.064 (0.843, 1.344)	0.600
HOMA-IR	1.287 (0.646, 2.563)	0.473

OR, odds ratio; CI, confidence interval; BMI, body mass index; WHR, waist-hip ratio; TG, triglycerides; HDL-C, high-density lipoprotein cholesterol; ALT, alanine aminotransferase; GGT, γ-glutaryl transferase; FINS, fasting insulin; HOMA-IR, homeostasis model assessment of insulin resistance

In correlation analyses, serum asprosin levels amounts had positive correlations with BMI (*r* = 0.288, *P* < 0.001), TG (*r* = 0.227, *P* = 0.001), GGT (*r* = 0.182, *P* = 0.007), Scr (*r* = 0.389, *P* < 0.001), FINS (*r* = 0.184, *P* = 0.006) and HOMA-IR (*r* = 0.142, *P* = 0.036), and a negative correlation with eGFR (*r*=-0.314, *P* < 0.001) (Table 3).

**Table 3:** Associations of serum asprosin levels with other clinical factors

Clinical data	R	*P*
Age	0.008	0.901
BMI	0.288	< 0.001
Time from T2DM diagnosis	0.068	0.314
SBP	-0.036	0.601
DBP	0.097	0.154
TC	0.010	0.881
TG	0.227	0.001
HDL-C	-0.109	0.108
LDL-C	0.021	0.758
ALT	0.116	0.087
AST	0.059	0.382
GGT	0.182	0.007
Scr	0.389	< 0.001
eGFR	-0.314	< 0.001
HbA1C	-0.063	0.354
FPG	-0.094	0.167
2hPG	-0.082	0.226
FINS	0.184	0.006
HOMA-IR	0.142	0.036
FC-P	-0.130	0.056

BMI, body mass index; SBP, systolic blood pressure; DBP, diastolic blood pressure; TC, total cholesterol; TG, triglycerides; HDL-C, high-density lipoprotein cholesterol; LCL-D, low-density lipoprotein cholesterol; ALT, alanine aminotransferase; AST, aspartate aminotransferase; GGT, γ-glutaryl transferase; Scr, serum creatinine; eGFR, estimated glomerular filtration rate; HbA1c, glycated hemoglobin; FPG, fasting plasma glucose; 2hPG, 2-hour postprandial glucose; FINS, fasting insulin; HOMA-IR, homeostasis model assessment of insulin resistance; FC-P, fasting C-peptide

## Discussion

In the present study, elderly adults with T2DM and MAFLD showed higher serum asprosin levels than those with T2DM but not MAFLD. Moreover, serum asprosin levels was independently associated with MAFLD in older diabetic adults. Therefore, simple blood draws to measure asprosin levels could be used to screen for MAFLD among patients with T2DM. Still, ultrasound is also an inexpensive, accurate, and readily available method for the screening of MAFLD. Maybe the two methods could be combined concurrently or sequentially in a strategy to improve the screening of MAFLD in the future.

In T2DM, asprosin levels are high and positively correlated with insulin resistance and could serve as a biomarker to predict T2DM onset and monitor treatment effects [17]. Additionally, high asprosin levels have been reported in T2DM [17], and asprosin levels correlated with FINS and HOMA-IR in the present study. Individuals with asprosin deficiency are extremely lean and maintain euglycemia despite low plasma insulin levels [26]. On the other hand, insulin resistance in patients and animal models is associated with high asprosin levels, and a single injection of recombinant asprosin in mice was sufficient to increase glycemia [26]. Asprosin also impairs insulin signaling [27] and causes inflammation, endoplasmic reticulum stress, and insulin resistance [28]. Regarding the markers of glucose and insulin metabolism, asprosin has a positive correlation with HOMA-IR and a negative association with HOMA-β [29]. Furthermore, asprosin levels are independently associated with T2DM [29–32]. Of note, You et al. [33] recently reported that asprosin is not necessarily elevated in all T2DM patients but mainly in those with peripheral artery disease, which warrants future investigation. In this work, asprosin levels were associated with BMI, TG, FINS, and HOMA-IR and inversely correlated with eGFR, indicating that high serum asprosin correlates with T2DM features, nephropathy, and metabolic syndrome.

In this study, asprosin levels were higher in older patients. Asprosin levels appear to increase with maternal age in pregnant women [34]. However, a study showed no correlation between age and asprosin levels [29]. This discrepancy could be due to different study populations. Nevertheless, obesity, T2DM, and MAFLD are conditions associated with age. The potential relationship between age and asprosin levels should be further investigated.

Asprosin has also been suggested to be associated with MAFLD [18, 19] and is considered a diagnostic marker of MAFLD [19]. Asprosin is independently associated with obesity [35–37] and blood TG levels [29, 37], which are two risk factors for MAFLD [14–16]. Asprosin is elevated in obese children and even more in counterparts with MAFLD [18]. The link between asprosin and MAFLD could be insulin resistance and inflammation [26, 38], as well as the endothelial-to-mesenchymal transition [33], but the exact mechanisms remain undefined. In the present work, individuals with T2DM and MAFLD had significantly elevated asprosin levels in comparison with T2DM cases without MAFLD.

Since early MAFLD is benign and reversible [39], timely diagnosis of MAFLD is important. In the present work, asprosin levels correlated with GGT levels, suggesting asprosin as a blood biomarker for diagnosing MAFLD. It would be easier to detect blood asprosin levels, which may be more accepted by the patients, compared with liver biopsy or more expensive imaging examinations. Asprosin could even be a treatment target since targeting asprosin with a specific antibody has been shown to improve hyperinsulinemia in metabolic syndrome [26, 40].

Asprosin also appears to be associated with T2DM complications such as diabetic nephropathy [41]. In addition to the association of asprosin with obesity [35–37], which is in turn associated with T2DM, high asprosin levels were also observed in polycystic ovarian syndrome (PCOS), a condition often diagnosed in association with obesity and T2DM [17]. Asprosin is also involved in the development of cardiovascular diseases (CVD) [17], and T2DM is a risk factor for CVD. Nevertheless, the exact causal relationships among the above factors and conditions remain to be investigated.

The current study had limitations. Firstly, because all patients were from only two health centers in the same city, a limited number of cases were analyzed, indicating low generalizability of the present findings. In addition, only older adults were included; consequently, more patients of different ages should be included. Secondly, in recent years, the same concept of disease rapidly evolved from non-alcoholic fatty liver disease [39] to MAFLD [24] to metabolic-associated steatotic liver disease (MASLD) [42], shedding some confusion in definitions and data analysis. Still, Hagstrom et al. [43] showed that 99.5% of the patients diagnosed with NAFLD met the criteria for MASLD, concluding that “previous natural history data can be used”. Thirdly, due to the cross-sectional design, no causal conclusions could be drawn, and whether higher asprosin causes or results from MAFLD deserves further attention. Fourthly, because liver biopsy is not popular among patients, the diagnosis of MAFLD was mainly made by ultrasound, which could miss some cases of mild hepatic MAFLD since the sensitivity of ultrasound is lower for mild MAFLD compared with more advanced diseases [21]. Fifthly, the relationship between serum asprosin and MAFLD severity could not be evaluated because of the diagnostic method used. Therefore, well-designed prospective and longitudinal trials are required to determine asprosin’s value in MAFLD screening and diagnosis in patients with T2DM. Sixthly, although the present study identified the asprosin as being independent from time from T2MD diagnosis, it has to be noted that the time from diagnosis is an imperfect metric to determine the duration of diabetes since some patients can have T2DM for some time before diagnosis. Still, it was the only metric available. Finally, the sample size was too small to perform a reliable analysis of asprosin’s diagnostic value.

## Conclusions

High serum asprosin levels are associated with MAFLD in patients with T2DM, after adjusted for age, time from T2DM diagnosis, WHR, TG, HDL-C, ALT, GGT, FINS, and HOMA-IR.

## Data Availability

All data generated or analyzed during this study are included in this published article.
